# Multimorbidity Patterns of Chronic Diseases among Indonesians: Insights from Indonesian National Health Insurance (INHI) Sample Data

**DOI:** 10.3390/ijerph17238900

**Published:** 2020-11-30

**Authors:** Atina Husnayain, Nopryan Ekadinata, Dedik Sulistiawan, Emily Chia-Yu Su

**Affiliations:** 1 Graduate Institute of Biomedical Informatics, College of Medical Science and Technology, Taipei Medical University, Taipei 106, Taiwan; atina.husnayain@mail.ugm.ac.id; 2 Center for Health Policy and Management, Faculty of Medicine, Public Health and Nursing, Universitas Gadjah Mada, Yogyakarta 55281, Indonesia; nopryan.ekadinata@mail.ugm.ac.id; 3 Faculty of Public Health, Universitas Ahmad Dahlan, Yogyakarta 55164, Indonesia; dedik.sulistiawan@ikm.uad.ac.id; 4 Clinical Big Data Research Center, Taipei Medical University Hospital, Taipei 110, Taiwan

**Keywords:** chronic disease, multimorbidity, public health informatics, claims data, Indonesia

## Abstract

Given the increasing burden of chronic diseases in Indonesia, characteristics of chronic multimorbidities have not been comprehensively explored. Therefore, this research evaluated chronic multimorbidity patterns among Indonesians using Indonesian National Health Insurance (INHI) sample data. We included 46 chronic diseases and analyzed their distributions using population-weighted variables provided in the datasets. Results showed that chronic disease patients accounted for 39.7% of total patients who attended secondary health care in 2015–2016. In addition, 43.1% of those were identified as having chronic multimorbidities. Findings also showed that multimorbidities were strongly correlated with an advanced age, with large numbers of patients and visits in all provinces, beyond those on Java island. Furthermore, hypertension was the leading disease, and the most common comorbidities were diabetes mellitus, cerebral ischemia/chronic stroke, and chronic ischemic heart disease. In addition, disease proportions for certain disease dyads differed according to age group and gender. Compared to survey methods, claims data are more economically efficient and are not influenced by recall bias. Claims data can be a promising data source in the next few years as increasing percentages of Indonesians utilize health insurance coverage. Nevertheless, some adjustments in the data structure are accordingly needed to utilize claims data for disease control and surveillance purposes.

## 1. Introduction

The rise of multimorbidities is commonly associated with large demands for healthcare utilization [1]. Therefore, studies of multimorbidities are common in the US and European countries. Unfortunately, this research area has not yet been well established in Asian countries [2]. The emerging issue of aging populations in several Asian countries has prompted an understanding of chronic multimorbidities. Most multimorbidity studies revealed that an advanced age is the factor most often correlated with increasing odds of having chronic multimorbidities [2,3,4,5,6,7], which corresponds to a large proportion of multimorbidities in elderly groups [8].

In fact, Indonesia is currently entering the early stages of an aging population with more than 25 million citizens currently aged ≥60 years [9] or 9% of the total population. Despite this condition, only limited programs for elderly groups that include health aspects can be provided due to the disbanding of the National Commission for the Elderly at the end of 2018 [10]. On the other hand, chronic disease and risk factor surveillance that should play key roles in tackling chronic multimorbidities has not become a major public health function [11]. Thus, even though researchers and policymakers already realize that there will be extensive demands for healthcare services in the next few years, chronic multimorbidity patterns have not been comprehensively explored.

One of the most recent nationally representative studies on chronic multimorbidities [12] was conducted in 2015. That study presented various patterns of chronic multimorbidities among Indonesian adults. That research used a self-reported method which may have caused some misclassification bias and affected the results to a certain degree. Moreover, it only included 15 chronic diseases and did not present actual chronic disease-related visits to healthcare facilities. Accordingly, in order to minimize limitations of the self-reported method, some analyses [2,5] also utilized claims data in presenting chronic multimorbidity patterns. It could be a better approach since comorbidity data are precisely recorded in both primary and secondary health care.

As claims data for research-related purposes are available from the Indonesian Health Insurance Agency (IHIA), this research explored chronic multimorbidity patterns among Indonesians. Analyses described the multimorbidity patterns that urgently need to be addressed particularly in countries with a developing rate of demographic aging, including Indonesia. A better understanding of chronic multimorbidity patterns might potentially facilitate improved planning of disease prevention and management programs [6], provide safer and more-effective healthcare delivery [5], and promote changes to classic disease paradigms [2]. This research can also possibly help in planning financial strategies as a significant financial burden is caused by multimorbidity cases [13].

Various chronic disease patterns in this research were derived from 1.7 million records of anonymous claims data recorded in 2015–2016 [14]. All provided datasets, including a referral care dataset (primary and secondary diagnoses), capitation- and non-capitation-based primary care datasets, and a membership dataset, were employed to depict numerous patterns across demographic characteristics and spatially distinctive disease dyads. Furthermore, this analysis also identified several advantages and challenges in utilizing Indonesian National Health Insurance (INHI) sample data as a complementary data source for chronic disease surveillance, prevention, and control programs.

## 2. Materials and Methods

This study used the second edition of the anonymous INHI sample data launched by the IHIA in August 2019 [14], and the research protocol has been reviewed with protocol number of KE/FK/1197/EC/2020. Datasets were derived from 1% of the membership coverage at the end of December 2016. Individual samples included in the dataset were sampled from primary healthcare (22,024 facilities) and family sampling frames (73,441,160 families). To increase the representativeness of the sample data, families were divided into three different strata, including families who received no medical treatment, families who received treatments in primary healthcare, and families who received medical treatments in both primary and secondary healthcare. Ten family samples were collected from each stratum using a stratified random sampling method. This process included 1,697,452 individual samples in the dataset. Sample data from this second edition provide five relational tables including membership dataset (1,697,452 members), capitation- (1,733,759 visits) and non-capitation-based primary care datasets (104,456 visits), referral care dataset (906,905 visits), and an additional table for secondary diagnoses (700,885 diagnoses) in referral care.

In this study, we utilized the referral care dataset (primary and secondary diagnoses) linked to the capitation- and non-capitation-based primary care datasets and a membership dataset in order to provide various chronic disease patterns. We included 46 chronic diseases summarized in 310 International Classification of Disease (ICD)-10 codes to collect chronic disease-related visits from a referral care table either from a primary or secondary diagnosis table. A list of ICD-10 codes used in this research was developed in a previous study by Koller and colleagues [15] and is provided in Supplementary Materials.

Patient IDs of chronic disease were traced back in the capitation- and non-capitation-based primary care datasets to their recorded diagnosis for chronic conditions. Demographic characteristics were then retrieved from the membership dataset along with spatially distinctive diagnoses from the referral care dataset. Data preprocessing was conducted using SAS EG 7.1 (SAS Institute, Cary, NC, USA). The analyses were weighted using individual weight variables provided in the datasets [14] which were determined from the family weighting. The family weighting was calculated based on the probability of the family being chosen as a sample in each stratum (primary healthcare and family strata). Weighting variables in the dataset included the family ID as the primary sampling unit, city as strata, and individual weighting for every single member.

Data visualization was performed using Tableau Public and Microsoft Power BI to illustrate various patterns of chronic multimorbidities. Findings are presented in butterfly charts, bar charts, line charts, and Sankey diagrams. Furthermore, prevalence odds ratios (PORs) were also used to assess risks of having chronic multimorbidities across regions. *p* values were not adjusted for multiple tests and are only to be interpreted in an exploratory manner. In order to provide comprehensive results, findings were then compared to multimorbidity patterns in other Asian countries. Lastly, this paper also discusses considerable advantages and challenges in utilizing INHI sample data as a complementary data source for chronic disease surveillance, prevention, and control programs.

## 3. Results

**Figure 1:**
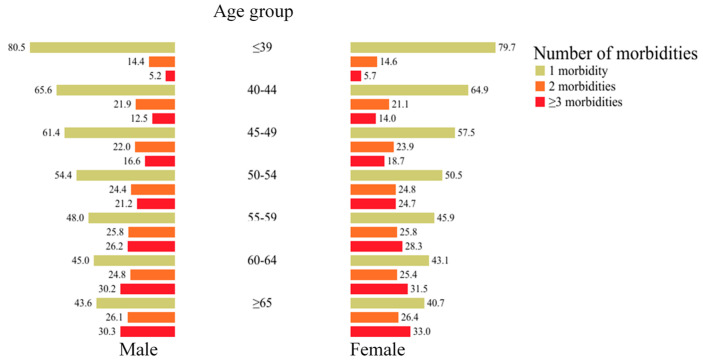
Weighted proportion of patients with chronic morbidities according to age group and gender.

**Figure 2:**
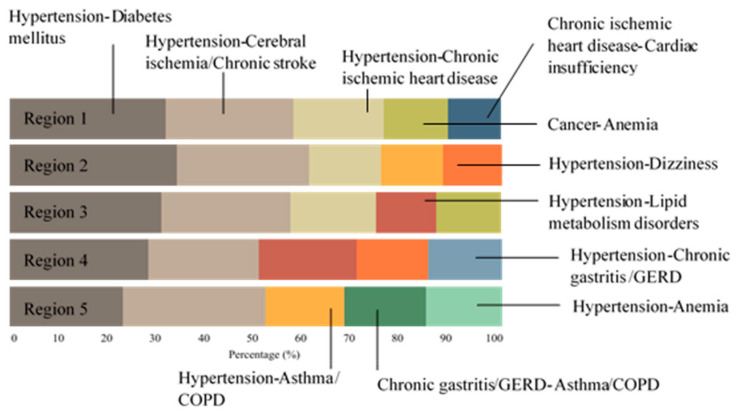
Proportions of the most common disease dyads according to region.

**Figure 3:**
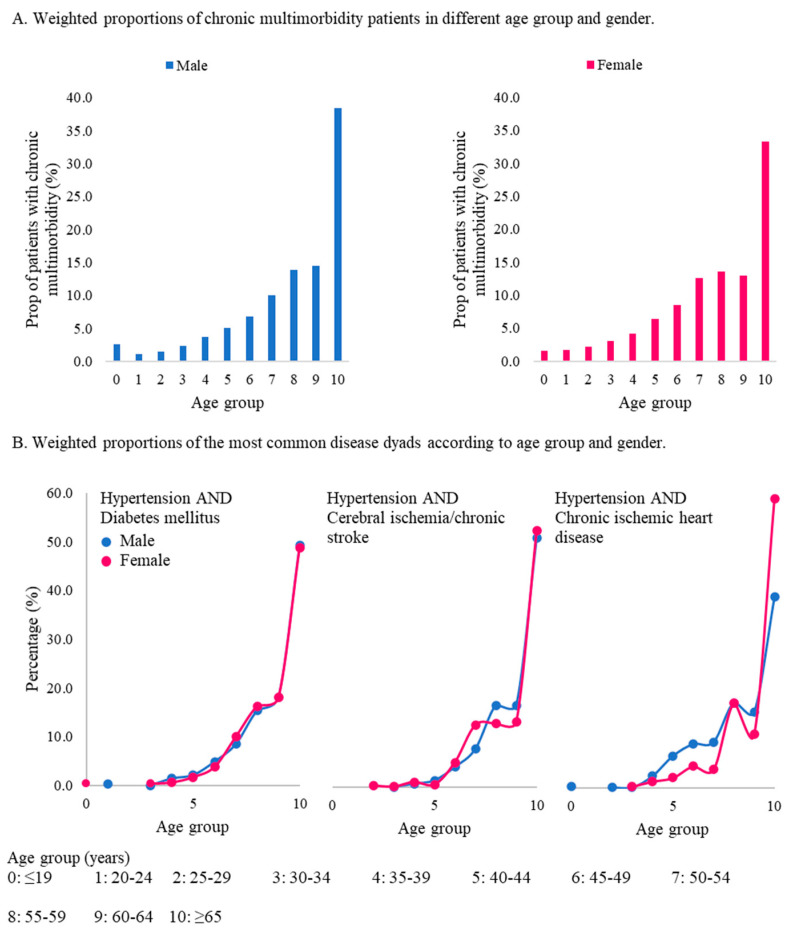
Weighted proportions of patients with chronic multimorbidities and the most common disease dyads according to age group and gender.

**Figure 4:**
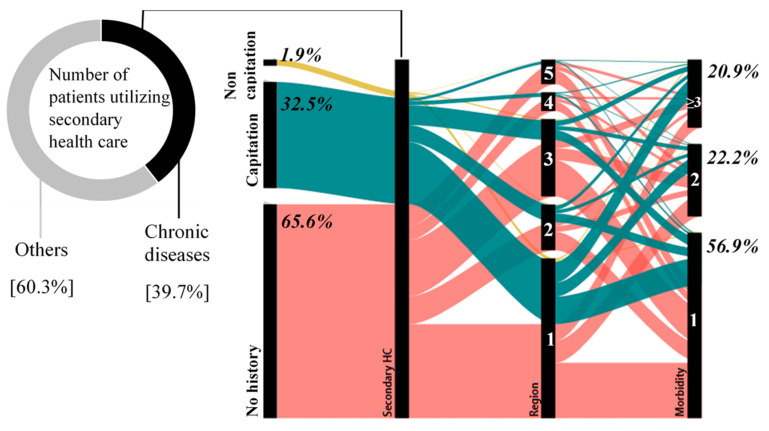
Patterns of healthcare utilization in chronic disease patients.

National health claims data identified 95,503 of 240,589 patients entering secondary health care as having chronic morbidities. This number accounted for 39.7% of healthcare utilization at the secondary level in 2015–2016. Furthermore, multimorbidity patterns across demographic characteristics and spatial distinctions are presented in Figure 1, Figure 2, Figure 3 and Figure 4 and Table 1 and Table 2.

### 3.1. Proportions and Distributions of Chronic Multimorbidities among Indonesians

The proportion of patients with chronic multimorbidities increased in the older age groups as shown in Figure 1. In the age group of ≤39 years, multimorbidities (two morbidities and three or more morbidities) only accounted for around 20%. But this number increased almost three times in the elderly group. This pattern seemed to be similar in both males (56.4%) and females (59.3%).

Moreover, spatially distinctive multimorbidity patterns were also described to understand the magnitude of the burden in all provinces as reported in Table 1. Based on this table, most patients with chronic conditions and multimorbidities were located on Java island with respective proportions of 60.0% and 63.8%. This is not a surprising finding since roughly 55% of recorded healthcare visits were from Java island. In terms of population density, 56.8% of the population lives on Java island.

### 3.2. Risk of Having Chronic Multimorbidities among Indonesians

Based on the POR calculations, age was the most important variable corresponding to a risk of having chronic multimorbidities. The age group of 40–44 years was two times more likely to have chronic multimorbidities than those aged ≤39 years. However, odds dramatically rose to five to six times in the elderly group (≥65 years old) in all regions.

The highest odds occurred in regions 3 and 4 in the age class of 45–49 years, signifying the presence of a risk of chronic multimorbidities in the younger age group. Moreover, region 5 also had the highest POR in the elderly group. These findings are opposite to those in Table 1, which only demonstrates a large proportion of chronic multimorbidities in region 1, since there is extensive healthcare utilization and a dense population in this region. In contrast, after dividing the country into five different regions, Table 2 implies circumstances of risks of chronic multimorbidities in other regions, beyond region 1.

Without controlling for age, persons who are female, currently married, and non-subsidy recipients were also identified as having a higher odds of multimorbidities compared to those in the reference group. However, gender presented non-statistically significant results in most regions. Nevertheless, in this study, a person who was currently married was 1.5 times more likely to have chronic multimorbidities than those who were not married. Lastly, non-subsidy recipients were also found to be more vulnerable to having chronic multimorbidities with odds of <1.5. Still, this may have been due to an extensive proportion of non-subsidy recipients which accounted for more than 70% of records. The non-subsidy recipient group is a group of insurance members who receives no insurance subsidies from the regional or central government. This group includes people who work for the government and the private sector and also have the financial ability to pay the monthly fee.

### 3.3. Common Disease Dyads and Disease Proportions in Different Age Groups and Genders

Figure 2 illustrates patterns of the most common disease dyads across the region. As pointed out in the graph, region 1 had a relatively similar pattern to that of region 3, and region 2 had a similar pattern to region 4, while region 5 had a quite different pattern. Hypertension was the leading disease that was present in 70% of the most common disease dyads in the five regions. The most common comorbidities with hypertension were diabetes mellitus (DM), cerebral ischemia/chronic stroke, and chronic ischemic heart disease.

Multimorbidity patterns were then identified across different ages and genders as presented in Figure 3 for the most common disease dyads. The proportion of female patients was higher than male patients in the age class of 20–54 years. Furthermore, increased proportions of patients were found in the older age group of ≥65 years in both the male (38.4%) and female groups (33.2%). Patterns of hypertension and DM seemed to be similar in both males and females. This disease dyad arose in females at ages of ≤19 years, and it did so at 20–24 years in the male group. Around 49% of cases were aged ≥65 years.

While hypertension and cerebral ischemia/chronic stroke are present in the 25–29-year age group in females and the 30–34-year age group in males, different from hypertension and DM, this disease dyad was increasing earlier and was higher in the female group starting from the age class of 45–49 years. Last, the disease dyad of hypertension and chronic ischemic heart disease occurred in the age group of ≤19 years in males and 30~34 years in females. The disease occurrences are increasing earlier and were higher in the male group beginning in the age group of 35–39 years. However, the proportion of this disease dyad dramatically increased at the age of ≥65 years in females. All disease dyads had increased proportions in the elderly group (≥65 years) following proportions of patients of more than one-third being aged ≥65 years.

### 3.4. Patterns of Healthcare Utilization for Chronic Disease Patients

Figure 4 illustrates patterns of healthcare utilization for chronic disease patients. From total patients who utilized secondary health care in 2015–2016, chronic disease patients accounted for 39.7% of total patients. Against that large number, 34.4% of chronic disease patients were also recorded utilizing health care at the primary level, both for capitation and non-capitation groups. In contrast, 65.6% of them were marked as having no history or were not recorded attending primary healthcare for chronic disease-related visits before visiting secondary healthcare. This finding indicates that chronic disease patients may contact healthcare facilities in a poor condition, and they were therefore directly referred to secondary health care.

In addition, this graph also shows that most chronic disease patients came from region 1 due to a high population density and large numbers of health facilities and healthcare utilization. Among chronic patients in secondary health care, those with chronic multimorbidities accounted for 43.1%. However, compared to the remaining patients who utilized secondary health care, those with chronic multimorbidities only accounted for 17.1% of total patients. This might have been caused by limited healthcare utilization due to inadequate healthcare coverage.

### 3.5. Potential Use of Claims Data for Chronic Disease Surveillance, Prevention, and Control Programs in Indonesia

In general, the use of claims data for surveillance purposes represents an important opportunity. The IHIA has been providing bridging facilities of the IHIA reporting system (PCare and INA CBGs) with health center information systems (Simpus) and hospital information systems. Therefore, any cases recorded in gatekeeper and referral care can be easily managed cross-platform for surveillance purposes, and as the number of healthcare facilities that contribute to the universal health coverage (UHC) system increases, data coverage will be elevated. However, there are several specific opportunities and challenges of utilizing claims data for chronic disease surveillance according to prior studies [2,5,17] (Table 3).

## 4. Discussion

This study successfully employed claims data to depict various patterns of chronic multimorbidities. Furthermore, based on findings presented in the “Results” section, some results need to be emphasized as follows.

1. *Age is the most important variable that corresponds to the risk of having chronic multimorbidities*. Numerous studies in Asian countries have also demonstrated an older age as the most common risk of having chronic multimorbidities [2,3,4,5,6,7], including a former study conducted in Indonesia [12]. A high proportion of multimorbidities in elderly groups shown in this study was also found in a previous study in a Chinese population [8]. Therefore, this fact could be a warning since Indonesia is currently undergoing development of demographic aging [9]. In addition, the highest odds occurred in regions 3 and 4 in the age class of 45–49 years, signifying the presence of chronic multimorbidity risks in that younger age group, which was also found in former studies [2,12]. These findings mean that in the next few years, a large burden of chronic multimorbidities will potentially result in massive healthcare needs with heavy cost burdens.
2. *Without controlling for age, persons who are female, currently married, and non-subsidy recipients were also identified as having higher odds of multimorbidities compared to those in the reference group.* This finding differs from previous research in Cambodia, Myanmar, Thailand, Vietnam [4], and Japan [5] that showed higher risks in male groups but resembled former studies in India [3,6], Indonesia [12], and Taiwan [2] that pointed out females as a vulnerable group for chronic multimorbidities. In addition, a higher chance of multimorbidities for married persons was also found in research of an Iranian population [18]. The correlation between the marital status and risk of multimorbidities is not completely understood; however, a previous study explained that this phenomenon might be associated with the presence of social support [3]. The risk might be biased by the large proportion of non-subsidy recipients in datasets, which accounted for more than 70% of records.
3. *Java island is the densest island for chronic multimorbidities*. This finding might be due to the highest coverage of health care being concentrated on Java. Differing from other regions, Java and Bali have lower ratios of primary and secondary healthcare facilities per 10,000 population which indicate a better availability of healthcare facilities [19]. This condition results in extensive case findings only in those five provinces and remaining low proportions in the 29 other provinces. Indeed, healthcare access directly affects healthcare utilization. Therefore, in this analysis, around 49% of chronic patients came from Java island or region 1. In terms of healthcare utilization at the primary level and number of patients with chronic multimorbidities, Java had higher respective proportions at 41.8% and 47.4% compared to non-Java areas (28.8% and 39.0%). This finding resembles a study in India which demonstrated that the highest proportion of multimorbid patients came from states with better access and utilization of healthcare services [6]. Hence, a lower proportion of multimorbidities might not represent the true condition in the population but could be caused by limited access and utilization of healthcare services. This result indicates that in the next few years, as health care coverage increases, the number of chronic patients will potentially increase in all provinces of Indonesia.
4. *Hypertension was the leading disease that was present in 70% of the most common disease dyads in the five regions.* The same hypertension pattern also occurred in other Asian countries, including Cambodia, Myanmar, Thailand, Vietnam [4], India [6], Japan [5], and an earlier study in Indonesia [12]. Similar patterns of comorbidities with hypertension (diabetes mellitus (DM), cerebral ischemia/chronic stroke, and chronic ischemic heart disease) were also present in Bangladesh [7] and Cambodia [4]. However, a previous study in Indonesia [12] illustrated different disease comorbidities including cardiac diseases, hypercholesterolemia, and arthritis. That study mentioned high proportions of hypertension in the female group in both adult (45.8%) and elderly groups (69.8%). That study also showed the proportions of cardiac disease of around 11.1% to 17.8% and low occurrences of DM (3.8%–5.2%) and stroke (0.3%–2.1%) [12]. Percentages of hypertension and cardiac diseases were higher in the elderly female group, while those of stroke and DM were greater in the elderly male group. Although patterns were only presented by disease proportions, it might be difficult to compare to the current analysis, which presents the pattern according to disease dyads.
5. *Over one-third (34.4%) of chronic disease patients were also recorded utilizing health care at the primary level, both for capitation and non-capitation groups.* As a consequence, in order to provide better healthcare services, primary health care should facilitate increasing awareness of multimorbidities [1].

On the other hand, compared to another study in Cambodia, Myanmar, Thailand, and Vietnam (72.6%) [4], the proportion of chronic multimorbidities in this analysis (43.1%) was lower but still higher than that in Taiwan (30.4%) [2]. Differences in multimorbidity rates might be caused by several factors, including the following.

1. *Access to healthcare services*. Better access and utilization of healthcare services might be dominant aspects of multimorbidity studies [6]. In addition, the health system may also affect health-seeking behaviors. Therefore, multimorbidities could be higher in countries that implement universal health coverage (UHC) systems with high levels of healthcare coverage, access, and utilization.
2. *The number of diseases used for the analysis*. Various lists of diseases selected to assess multimorbidities may cause different magnitudes of multimorbidities [2]. Previous studies in Asian countries used five to 22 types of chronic diseases [2,3,4,5,6,7,12]. To obtain better representativeness of multimorbidities, this research used 46 chronic diseases. However, a larger scope of diseases might have expanded the analysis.
3. *Rate of demographic aging*. According to research in Taiwan [2], the rate of demographic aging may also affect the magnitude of multimorbidities. Therefore, multimorbidity rates might be higher in countries with high rates of demographic aging.
4. *Age group used in the analysis*. Most multimorbidity studies examined adults [3,6,7,12] and elderly groups [5]. Research on multimorbidities is rarely done for all ages [2,4]. Therefore, the rate of chronic multimorbidities commonly differs across studies.
5. *Data source utilized for the analysis*. Survey data obtained through a self-reported method [3,4,6,7,12] are the most common data source for chronic multimorbidity studies. Besides that, there are a few studies that utilized claims data [2,5] and surveillance data [17]. Accordingly, different data sources with diverse data collection methods could affect rates of multimorbidities at a certain level.

Given the emerging burden of chronic multimorbidities in Asian countries, chronic disease and risk factor surveillance has not yet become a major public health function [11]. For example, Indonesia conducted a STEP-wise approach to chronic disease risk factor surveillance only in certain years including in 2001, 2003, and 2006 [20]. These were national (2001) and sub-national (2003, 2006) surveys to collect preventable risk factors for non-communicable diseases in rural Indonesia. Another survey that also collects risk factors on non-communicable diseases is the Indonesian Family Life Survey (IFLS) [21]. The IFLS is an ongoing longitudinal survey that includes over 30,000 individuals living in 13 provinces in Indonesia. The survey was conducted in 1993, 1997, 2000, 2007, and 2014. However, there is no sentinel survey or surveillance of chronic morbidities that can be generated every year.

In addition, the Indonesian government through the Ministry of Health also offers a primary care-based program for early detection and monitoring of risk factors for non-communicable diseases called Posbindu [22]. The number of community groups involved in the Posbindu program has reached 33,679 [23], but it still suffers from low involvement (at 25%) of elderly populations due to inadequate facilities, physical disabilities, and communication problems [22]. Moreover, the IHIA as a public institution that corresponds to implementation of UHC in Indonesia also launched a program called Prolanis. This is a chronic disease management program that is also centered on primary health care [24] and is dedicated to type 2 diabetes and hypertension patients that are registered in the Indonesian health insurance system. However, implementation of this program is still limited due to various barriers encountered during its implementation [25].

These conditions illustrate that there is no stable chronic disease control program currently being conducted in Indonesia or data sources for surveillance purposes. Therefore, in this study we utilized claims data to assess their capacity to be used as a complementary data source for chronic disease surveillance, prevention, and control programs. Compared to the survey method, claims data can more economically present many kinds of disease patterns and are not influenced by recall bias. Nevertheless, claims data do not contain disease severity data, or information on laboratory results and out-of-pocket expenditures, since claims data are generated for cost purposes. Claims data also might be affected by misclassification errors in diagnosis that cannot be traced back to the source. As a newly published dataset, INHI sample data cover a short period of time and are not as complete as other established claims data, such as the National Health Insurance Research Database from Taiwan that includes multiple aspects in datasets [26].

Despite these limitations, using claims data as the data source for chronic disease surveillance may be promising in the next few years. A surveillance system can be built like the chronic disease surveillance system in Canada [17], which was built using three different databases. That system was constructed using hospital records, physician billing claims, and population registry databases that are linked using health insurance numbers. The same system can also be developed in Indonesia using a cohort of claims data, derived from 1% of the population. In order to get a better understanding of demographic patterns, claims data can be linked to a population registry using residence numbers as a unique identifier.

Moreover, prevention and control programs need to be implemented in primary health care since chronic multimorbidities can be identified at the primary level as shown in this research’s findings. Multimorbidities also need to be considered when planning disease prevention and management programs [6]. Thus, national management program such as Prolanis should be designed based on disease multimorbidities.

## 5. Conclusions

The INHI sample data can be used to depict chronic multimorbidity patterns among Indonesians. Chronic disease patients accounted for 39.7% of total patients who utilized secondary health care in 2015–2016, and 43.1% of those were identified as having chronic multimorbidities. Multimorbidities are strongly correlated with an advanced age, with large numbers of patients and visits in all provinces, beyond those on Java island. In addition, hypertension was the leading disease that was present in 70% of the most common disease dyads in five regions. The most common comorbidities with hypertension were diabetes mellitus, cerebral ischemia/chronic stroke, and chronic ischemic heart disease. Lastly, proportions of certain disease dyads differed according to age group and gender.

Compared to the survey method, claims data can more economically present many kinds of disease patterns and are not influenced by recall bias. Using claims data as a data source for chronic disease surveillance may be promising in the next few years. Nevertheless, claims data are generated for cost purposes, and some adjustments in data structures are accordingly needed.

## Figures and Tables

**Table 1:** Distributions of the numbers of patients with chronic conditions and multimorbidities.

No.	Island	Province	No. of Patients with Chronic Conditions	No. of Patients with Chronic Multimorbidities	Total Population
1	Sumatera	Aceh	3208	1187	4,993,385
2		North Sumatera	4538	1695	13,923,262
3		Riau	1675	706	6,330,941
4		West Sumatera	2708	1251	5,190,577
5		Jambi	1265	473	3,397,164
6		Bengkulu	751	254	1,872,136
7		South Sumatera	2064	760	8,043,042
8		Lampung	1954	669	8,109,601
9		Island of Riau	735	270	1,968,313
10		Island of Bangka Belitung	535	217	1,370,331
11	Java	Banten	3483	1443	11,934,373
12		West Java	17,273	7791	46,668,214
13		Central Java	15,149	7277	33,753,023
14		Special Region of Jakarta	6256	2825	10,154,134
15		Special Region of Yogyakarta	2478	1300	3,675,768
16		East Java	12,636	5623	38,828,061
17	Kalimantan	West Kalimantan	1208	501	4,783,209
18		Central Kalimantan	727	333	2,490,178
19		South Kalimantan	1043	440	3,984,315
20		East Kalimantan	1639	621	3,422,676
21		North Kalimantan	341	131	639,639
22		West Sulawesi	352	151	1,279,994
23		Central Sulawesi	986	428	2,872,857
24	Sulawesi	Gorontalo	531	225	1,131,670
25		North Sulawesi	1319	551	2,409,921
26		South Sulawesi	3580	1355	8,512,608
27		Southeast Sulawesi	650	243	2,495,248
28	Maluku	North Maluku	265	112	1,160,275
29		Maluku	483	194	1,683,856
30	Nusa	Bali	1607	569	4,148,588
31	Tenggara	West Nusa Tenggara	1282	517	4,830,118
32		East Nusa Tenggara	1394	571	5,112,760
33	Papua	West Papua	978	351	868,819
34		Papua	411	156	3,143,088
		**Total**	**95,504**	**41,190**	**255,182,144**

Note: Numbers of patients with chronic conditions and multimorbidities were collected from INHI sample data analyzed in this study, while the total population numbers were gathered from a 2015 population survey provided by the Indonesian Central Bureau of Statistics [16].

**Table 2:** Risks of having chronic multimorbidities according to region.

Characteristics		Overall	POR by Region				
			1	2	3	4	5
Gender	Male	Reference					
	Female	1.0	1.0	1.0 ^ns^	1.0 ^ns^	1.0 ^ns^	1.0 ^ns^
Age group (years)	≤39	Reference					
	40−44	2.2	2.2	2.5	2.0	2.5	2.0
	45−49	2.9	2.9	2.7	3.1	3.0	2.7
	50−54	4.0	4.1	3.6	3.8	3.7	4.1
	55−59	4.8	4.9	4.5	4.6	4.7	3.6
	60−64	5.4	5.7	5.4	4.3	5.8	5.5
	≥65	6.1	6.2	6.1	5.4	5.6	6.5
Marital status	Currently not married	Reference					
	Currently married	1.6	1.6	1.6	1.6	1.5	1.6
Type of membership	Subsidies’ recipients	Reference					
	Non subsidies’ recipients	1.3	1.2	1.3	1.3	1.0 ^ns^	1.2
Legend:							
Prevalence odds ratio (POR)		0–2	2.1–3.1	3.2–4.1	4.2–5.4	5.5–6.5	

Note: all PORs were statistically significant at *p* ≤ 0.05, except cells marked with ns (non-significant). Regions are defined by the government for cost purposes. Region 1: Banten, Jakarta, West Java, Central Java, Yogyakarta, East Java; Region 2: West Sumatera, Riau, South Sumatera, Lampung, Bali, West Nusa Tenggara; Region 3: Aceh, North Sumatera, Jambi, Bengkulu, Bangka Belitung, Island of Riau, West Kalimantan, North Sulawesi, Central Sulawesi, South East Sulawesi, West Sulawesi, South Sulawesi, Gorontalo; Region 4: South Kalimantan, East Kalimantan, North Kalimantan, Central Kalimantan; Region 5: East Nusa Tenggara, Maluku, North Maluku, Papua, West Papua.

**Table 3:** Opportunities and challenges of utilizing Indonesian National Health Insurance sample data as a complementary data source for chronic disease surveillance.

No.	Opportunities	Challenges
1.	Presents patterns across various aspects including demographic characteristics, time, spatial distinctions, disease dyads, and claim-related variables	Contains no disease severity data or information on laboratory results
2.	More economical than primary data collection through a survey	Does not record out of pocket expenditures
3.	Not influenced by recall bias	May be affected by misclassification errors of diagnoses

## Supplementary Materials

The following are available online at https://www.mdpi.com/1660-4601/17/23/8900/s1, Table S1: List of chronic conditions included in the analysis.
