# Supplementary material for: Multimorbidity Patterns of Chronic Diseases among Indonesians: Insights from Indonesian National Health Insurance (INHI) Sample Data
Source: Int J Environ Res Public Health. 2020 Nov 30;17(23):8900. doi: 10.3390/ijerph17238900 (PMC7731032; doi:10.3390/ijerph17238900)
Supplement: Supplementary file 1 [file ijerph-17-08900-s001.pdf]

Table S1: List of chronic conditions included in the analysis.

No.	Chronic condition	ICD-10 codes
1	Hypertension	I10–I15
2	Lipid metabolism disorders	E78
3	Chronic low back pain	M40–M45, M47, M48.0–M48.2, M48.5–M48.9 M50–M54
4	Severe vision reduction	H17–H18, H25–H28, H31, H33, H34.1–H34.2, H34.8–H34.9, H35–H36, H40, H43, H47, H54
5	Joint arthrosis	M15–M19
6	Diabetes mellitus	E10–E14
7	Chronic ischemic heart disease	I20, I25, I21
8	Thyroid diseases	E01–E05, E06.1–E06.3, E06.5, E06.9, E07
9	Cardiac arrhythmias	I44–I45, I46.0, I46.9, I47–I48, I49.1–I49.9
10	Obesity	E66
11	Hyperuricemia/gout	E79, M10
12	Prostatic hyperplasia	N40
13	Lower limb varicosis	I83, I87.2
14	Liver disease	K70, K71.3–K71.5, K71.7, K72.1, K72.7, K72.9, K73–K74, K76
15	Depression	F32–F33
16	Asthma/COPD	J40–J45, J47
17	Gynecological problems	N81, N84–N90, N93, N95
18	Atherosclerosis/PAOD	I65–I66, I67.2, I70, I73.9
19	Osteoporosis	M80–M82
20	Renal insufficiency	N18–N19
21	Cerebral ischemia/chronic stroke	I60–I64, I69, G45
22	Cardiac insufficiency	I50
23	Severe hearing loss	H90, H91.0, H91.1, H91.3, H91.8, H91.9
24	Chronic cholecystitis/gallstones	K80, K81.1
25	Somatoform disorders	F45

No.	Chronic condition	ICD-10 codes
26	Hemorrhoids	I84
27	Intestinal diverticulosis	K57
28	Rheumatoid arthritis/chronic polyarthritis	M05–M06, M79.0
29	Cardiac valve disorders	I34–I37
30	Neuropathies	G50–G64
31	Dizziness	H81–H82, R42
32	Dementia	F00–F03, F05.1, G30, G31, R54
33	Urinary incontinence	N39.3–N39.4, R32
34	Urinary tract calculi	N20
35	Anemia	D50–D53, D55–D58, D59.0–D59.2, D59.4–D59.9, D60.0, D60.8, D60.9, D61, D63–D64
36	Anxiety	F40–F41
37	Psoriasis	L40
38	Migraine/chronic headache	G43, G44
39	Parkinson's disease	G20–G22
40	Cancer	C00–C14, C15–C26, C30–C39, C40–C41, C43–C44, C45–C49, C50, C51–C58, C60–C63, C64–C68, C69–C72, C73–C75, C81–C96, C76–C80, C97, D00–D09, D37–D48
41	Allergy	H01.1, J30, L23, L27.2, L56.4, K52.2, K90.0, T78.1, T78.4, T88.7
42	Chronic gastritis/GERD	K21, K25.4–K25.9, K26.4–K26.9, K27.4–K27.9, K28.4–K28.9, K29.2–K29.9
43	Sexual dysfunction	F52, N48.4
44	Insomnia	G47, F51
45	Tobacco abuse	F17
46	Hypotension	I95
